# Design, Production, Characterization, and Use of Peptide Antibodies

**DOI:** 10.3390/antib12010006

**Published:** 2023-01-13

**Authors:** Nicole H. Trier, Gunnar Houen

**Affiliations:** 1 Department of Neurology, Rigshospitalet Glostrup, Valdemar Hansens Vej 13, 2600 Glostrup, Denmark; 2 Department of Biochemistry and Molecular Biology, University of Southern Denmark, Campusvej 55, 5223 Odense, Denmark

Antibodies are key reagents in diagnostics, therapeutics, and experimental biology, capable of detecting numerous targets. The immunization of animals (rabbits, mice, etc.) with antigens mixed with an adjuvant and the induction of antibodies is a well-established process. This procedure for the generation of monoclonal antibodies was originally described by Köhler and Milstein, and is well-known and has been thoroughly tested [1,2,3,4,5]. Proteins were originally the focus for antibody production; however, in cases where the native protein is not available or modified targets are of interest, synthetic peptides coupled with an immunogenic carrier can be used as antigens [2,3,4,5,6,7].

The choice of suitable peptides is crucial for the generation of a good peptide antibody. The peptides used are typically 10–20 amino acids long and can be designed to represent many different targets, e.g., post-translational modifications, terminal ends, areas of high conservation, turns, loops, α-helices, etc. [2,3,5]. Based on this, peptide antibodies have many applications, especially because they can easily be produced against multiple targets [2,3,4,5,6,7,8,9,10,11]. For example, mutation-specific peptide antibodies have become important research and diagnostic tools in malignant and premalignant conditions [5,9,11].

In addition to peptide antibodies obtained by the immunization of animals, they can be produced using mammalian expression systems, which facilitate native antibody folding and post-translational modifications [12]. This approach typically employs the co-transfection of heavy chain (HC) and light chain (LC) genes on separate plasmids [12,13], although the use of a bidirectional vector encoding both genes on a single plasmid may be applied, as recently described by Carrara et al. [14]. Approaches such as these may enable efficient small-scale antibody production by transient cell transfection. Another well-established method is phage display, based on the genetic engineering of bacteriophages and repeated rounds of antigen-guided selection and phage propagation [15,16]. This approach was recently applied by Anderson et al. to generate antibodies specific for a Lassa virus protein [16]. Crucial in this approach is the selection of phage-displayed antibody libraries using synthetic peptides as targets, resulting in the generation of peptide-specific antibodies, commonly referred to as phage-specific peptide antibodies [15]. Using phase display, the in vitro production of antibodies to virtually any target is feasible.

Antibodies are typically expressed in a basic format with two HCs and two LCs (HC_2_LC_2_) linked via disulfide bonds, although some antibodies are occasionally prone to dimerization, or even oligomerization, as recently described by Mieczkowski et al. [17,18]. However, other antibody formats exist [4,13,15], for example, Anderson et al. recently described the production of a single-domain antibody consisting of a monomeric variable antibody domain which may have diagnostic and therapeutic potential [16]. Another example includes a recently published model developed by Ramasubramanian et al., who described a bivalent IgG where two different variable HC domains with distinct binding specificities were grafted onto the first constant HC and constant LC domains, resulting in an antibody with dual specificity [13]. Antibodies composed only of two HCs are commonly found in sharks and camelids, and were recently produced by Mieczkowski et al., showing good physiochemical properties and stability [17].

Independent of antibody design and production method, relevant antibodies should be identified by examining binding to the respective targets in the assay, where they are intended to be used, in order to avoid assay restrictions [2,3,19,20]. For example, peptide antibodies do not necessarily recognize native structures or do not necessarily detect the final target in all immunoassay formats [3,19,20]. Thus, it is essential to test the produced antibodies in a variety of formats, and ideally in the format of the intended use.

Following selection, (peptide) antibodies should be characterized in terms of reactivity, specificity, and cross-reactivity, e.g., by titration assays and competition studies, where the relevant target is used as an inhibitor [2,3,5]. Moreover, biochemical characteristics such as isotype/subtype, solubility, stability, and binding characteristics should be determined [3]. Antibodies may be characterized systematically in relation to the antigenic epitope by various mapping techniques [2,3,5,21]. For antibodies recognizing a conformational epitope, this may involve mass spectrometry, nuclear magnetic resonance, X-ray crystallography, and surface plasmon resonance, as described by Ramasubramanian et al., Anderson et al., and Mieczkowski et al.; however, these are time-consuming approaches [13,16,18]. In contrast, if the epitope of the respective antibody is continuous, which typically applies to peptide antibodies, but also to some antibodies generated for full-length proteins or protein fragments [5,21], antibodies are more easily characterized by epitope mapping or alternatively by analyzing antibody reactivity to peptides with sequence homology, as recently described by Bergmann et al. and Fanelli et al., respectively [21,22]. Here, amino acids essential for antibody binding are typically identified by Ala scanning and “functionality” scanning after the terminal “borders” crucial for antibody reactivity have been identified using systematically N- and C-terminally truncated peptides [5,21,23]. For the characterization of peptide antibodies, resin-bound peptides have been applied with success, because this approach is rapid and saves time compared with the use of free peptides [5,21,23]. The advantage of using resin-bound peptides is that they may be tested for binding activity directly on a solid support [5,21,23], where the peptide is connected to the resin through the -OH group of the C-terminal amino acid. This allows the peptide to fold into a conformation resembling the native conformation, whereas during the coating of free peptides on a solid surface, the peptide may change conformation, or crucial amino acid side chains necessary for the detection of antibody binding may be masked [3,19].

Peptide antibodies are used for research and diagnostics in many different immunoassays, e.g., immunohistochemistry, immunocytochemistry, immunoblotting, immunoprecipitation, and sandwich assays [5,8,9,10,11,15,24]. In the diagnostic field, peptide antibodies may aid in the diagnosis of diseases and infections [8,9,11]. For example, peptide antibodies have been used in immunohistochemistry for the detection of specific cancerous point mutations with great success, such as peptide antibodies specific for point mutations or deletions in B-raf, isocitrate dehydrogenase, and epidermal growth factor receptor, which are associated with melanoma, lung adenocarcinoma, and gliomas, respectively [9,11]. Moreover, a peptide antibody to the frame-shifted C-terminal of calreticulin was recently described; it showed promising results as a diagnostic candidate for calreticulin-associated myeloproliferative disorders [5]. Additionally, peptide antibodies have been used for detecting other tumors [10,11,15]. Moreover, peptide antibodies have been used for diagnosing neurodegenerative diseases, disorders of the immune system, infectious diseases, cardiovascular diseases, and other disorders, and to detect the presence of various viruses, bacteria, and parasites [8,11,25]. Despite obvious potential, the clinical therapeutic use of peptide antibodies still needs to be demonstrated. A promising example is peptide antibodies to gp120, which have been shown to neutralize HIV-1 infectivity in vitro [6]. Similar, a peptide antibody to procathepsin D has been proposed to inhibit breast cancer development, and treatment with a peptide antibody to apoB has been shown to reduce atherosclerosis in hypercholesterolemic mice, indicating that this antibody has therapeutic potential [26,27]. Finally, a peptide antibody to C5a, a protein fragment released from the cleavage of complement component C5 by the protease C5-convertase, has shown promising results in inhibiting sepsis [24]. Collectively, the abovementioned examples indicate that peptide antibodies have an unresolved potential in the therapeutic field.

The seven articles featured in this Special Issue, addressing the design, production, characterization, and use of (peptide) antibodies, are of considerable interest to scientists investigating approaches related to this topic.
